# Complementary and alternative medicine for the treatment of bronchiolitis in infants: A systematic review

**DOI:** 10.1371/journal.pone.0172289

**Published:** 2017-02-17

**Authors:** Kok Pim Kua, Shaun Wen Huey Lee

**Affiliations:** 1 School of Pharmacy, Monash University, Bandar Sunway, Selangor, Malaysia; 2 Department of Pharmacy, Petaling District Health Office (Ministry of Health), Kelana Jaya, Selangor, Malaysia; University of Alberta, CANADA

## Abstract

**Background:**

Bronchiolitis is a common cause of hospitalization among infants. The limited effectiveness of conventional medication has prompted the use of complementary and alternative medicine (CAM) as alternative or adjunctive therapy for the management of bronchiolitis.

**Aims:**

To determine the effectiveness and safety of CAM for the treatment of bronchiolitis in infants aged less than 2 years.

**Methods:**

A systematic electronic search was performed in Medline, Embase, CINAHL, AMED, and Cochrane Central Register of Controlled Trials (CENTRAL) from their respective inception to June 30, 2016 for studies evaluating CAM as an intervention to treat bronchiolitis in infants (1 month to 2 years of age). The CAM could be any form of treatment defined by the National Center for Complementary and Integrative Health (NCCIH) and was utilized either as a single agent or adjunctive therapy. The predefined primary outcome was length of hospital stay. Secondary outcomes were time to resolution of bronchiolitis symptoms, adverse events, and all other clinical outcomes reported by the included studies.

**Results:**

The review identified 11 studies (8 randomized controlled trials and 3 cohort studies) examining four herbal preparations and four supplements used either as adjunctive or alternative therapy for bronchiolitis in 904 infants. Most studies were of moderate quality. Among six studies reporting on length of stay, a significant benefit was found for Chinese herbal medicine compared to ribavirin in one cohort study (n = 66) and vitamin D compared to placebo in one randomized controlled trial (n = 89). Studies of Chinese herbal medicine (4 studies, n = 365), vitamin D (1 study, n = 89), N-acetylcysteine (1 study, n = 100), and magnesium (2 studies, n = 176) showed some benefits with respect to clinical severity scores, oxygen saturation, and other symptoms, although data were sparse for any single intervention and the outcomes assessed and reported varied across studies. Only five studies reported on adverse events; no serious adverse events were reported.

**Conclusions:**

Among 11 studies examining the effect of CAM on inpatients with bronchiolitis, six reported on the review’s primary outcome of length of hospital stay. In general, findings did not show a significant benefit associated with the primary outcome. Preliminary evidence indicated that Chinese herbal medicine mixtures, vitamin D, N-acetylcysteine, and magnesium might be useful in managing the symptoms of bronchiolitis. However, the evidence was not sufficient or rigorous enough to formulate recommendations for the use of any CAM. Among studies that reported adverse events, no serious harms were noted.

## Introduction

Bronchiolitis is the most common acute lower respiratory tract infection of viral origin among infants [1]. It is characterized by cough, rhinorrhea, crackles, wheezes, fever, and hypoxemia [2–4]. Common etiology includes respiratory syncytial virus, rhinovirus, adenovirus, coronavirus, human metapneumovirus, influenza, or parainfluenza [5, 6]. The economic and social implications of bronchiolitis are substantial. In North America, hospitalizations attributable to bronchiolitis have increased twofold over the past two decades [7, 8]. The disease currently accounts for more than 100 thousand hospital admissions annually at an estimated cost of $1.73 billion [9]. In the United Kingdom, 1 in 3 infants will develop bronchiolitis in the first year of life and 2 to 3% of all infants require hospital admissions. In England alone, there were 30,451 secondary care admissions for bronchiolitis between 2011 and 2012 [10].

Despite decades of research, effective pharmacotherapy for bronchiolitis is still lacking. The current treatment is controversial and there are no definitive recommendations for the use of any drug in the routine management of bronchiolitis [6]. Common pharmacological agents such as bronchodilator, corticosteroid, and hypertonic saline have been shown to only provide symptomatic relief [11]. Maintaining hydration and oxygenation of patient remains the cornerstone of management [12]. In the absence of efficacious curative therapy, the use of complementary and alternative medicine (CAM) in bronchiolitis is gaining popularity [13].

Over the past few years, several studies have examined the effects of different CAMs for bronchiolitis, but making sense of this literature requires a comprehensive and systematic synthesis of the available evidence. The aim of the current study was to comprehensively appraise the effectiveness and safety of CAMs for the treatment of bronchiolitis in infants.

## Methods

This study was conducted and reported following the process as specified in the PRISMA statement [14].

### Search strategy and selection criteria

A comprehensive electronic literature search was carried out in Medline, Embase, CINAHL, AMED, and CENTRAL from inception until June 30, 2016 by one investigator (KPK). The search strategy was constructed using a combination of keywords including “alternative medicine”, “complementary medicine”, “herbal medicine”, herb*, “traditional medicine”, “chinese medicine”, “chinese traditional”, “asian traditional”, “alternative therapy”, “complementary therapy”, “herbal therapy”, “traditional therapy”, supplement*, antioxidant, vitamin, natural, homeopathy, kinesiology, kinesiotherapy, “osteopathic medicine”, “osteopathic manipulation”, and bronchiolitis (S1 Text). This was complemented with additional searches of bibliographies in relevant primary and review articles.

Articles were included if they met the following criteria: (1) randomized controlled trials or prospective cohort studies published in English or Chinese language and available in full-text; (2) investigated the use of any form of complementary and alternative medicine defined by the National Center for Complementary and Integrative Health (NCCIH) as a group of diverse medical and health care systems, practices, and products that are not generally considered part of conventional medicine [15] either as a single agent or adjunctive therapy; (3) had a comparison group (either placebo or other therapy); and (4) involved inpatients or outpatients aged one month to two years with clinically diagnosed bronchiolitis [16].

### Study selection and data extraction

Two investigators (KPK and SWHL) independently screened the titles and abstracts. Full texts of relevant articles were retrieved and reviewed independently to determine eligibility for inclusion in the review. Any disagreement was resolved through discussion.

Data regarding study design, participants, interventions, clinical outcomes, and adverse events were independently abstracted by two investigators (KPK and SWHL) using a standardized data collection form. We also contacted five corresponding authors for additional information [17–21] and two responses were received [17, 20].

### Study quality assessment

Two investigators (KPK and SWHL) independently assessed the methodological quality of randomized controlled trials using Cochrane risk of bias assessment tool, which covered seven domains: random sequence generation (selection bias), allocation concealment (selection bias), blinding of participants and personnel (performance bias), blinding of outcome assessors (detection bias), incomplete outcome data (attrition bias), selective reporting (reporting bias) and an auxiliary domain (other bias) [22]. For cohort studies, Newcastle-Ottawa Quality Assessment Scale was used, which evaluated the study methodologies based upon three parameters: selection of participants, comparability of cohorts on the basis of design or analysis, and outcome ascertainment. Disagreements were resolved by consensus between two investigators (KPK and SWHL) [23].

### Data analysis

The predefined primary outcome was length of hospital stay. Secondary outcomes included time to resolution of bronchiolitis symptoms, adverse events, and all other clinical outcomes reported by the included studies such as clinical severity scores, rate of cure, oxygen saturation, bronchiolitis symptomatology presentation over the treatment period, duration of fluid or nutrition replacement, use of oxygen supplementation, and use of respiratory support.

Characteristics and results of all included studies were summarized and tabulated. Descriptive analysis was performed for all studies by both investigators (KPK and SWHL), and the results were presented narratively. Data were extracted to calculate relative risk (RR) for dichotomous outcomes or mean difference (MD) for continuous outcomes and their associated 95% confidence intervals (CIs) and p-values with a random effects model using Review Manager (RevMan) software, version 5.3. For studies that reported the data as medians, the respective median values and p-values were presented accordingly. A probability of ≤0.05 was considered statistically significant [24–27]. A meta-analysis was not performed due to the variability in the different outcomes assessed, study designs, and CAM interventions used.

## Results

### Study characteristics

**Fig 1:**
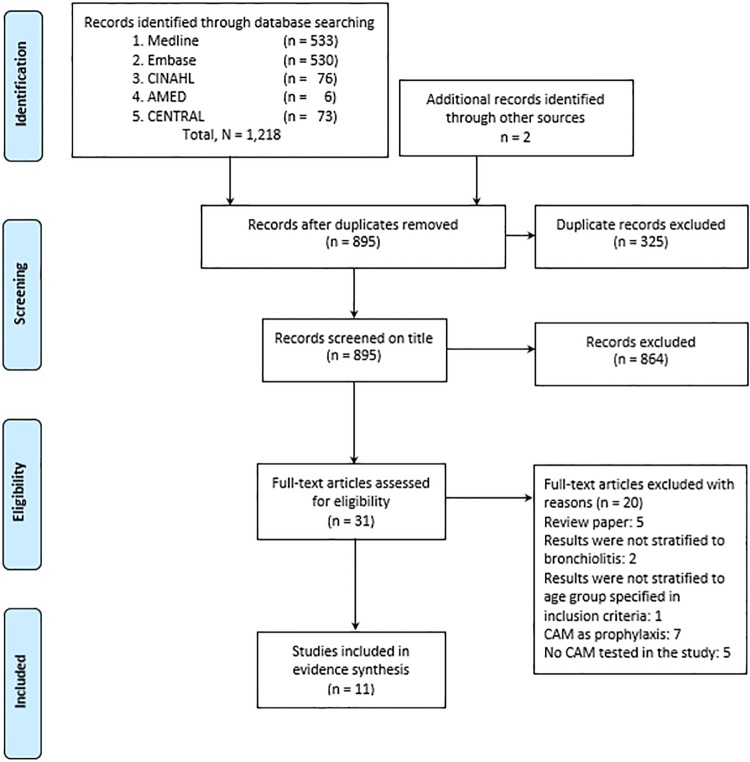
Preferred Reporting Items for Systematic Reviews and Meta-analyses (PRISMA) flow diagram outlining study screening, identification, inclusion and exclusion.

Our initial search yielded 1,220 studies, of which 31 underwent full-text evaluation and 11 unique studies were included in this review (Fig 1). They comprised 8 randomized controlled trials [17–21, 28–30] and 3 cohort studies [31–33] conducted in Asia, enrolling a total of 904 inpatient infants presenting with bronchiolitis. Sample size of the studies varied between 24 and 133 participants, with age ranging from 1 to 24 months. The studies were published between 1997 and 2015 in English (n = 9) [17–21, 28–30, 32] and Chinese (n = 2) [31, 33] language. Four studies were multicenter [17, 18, 28, 30] and seven were single-center [19–21, 29, 31–33]. Study duration ranged from one day to one week. In all studies, bronchiolitis was diagnosed by a physician based on symptoms of acute wheezing or respiratory distress. In seven studies, the infants were diagnosed as first time wheezers [17–19, 21, 28–30]. Another one study recruited a combination of wheezing and non-wheezing patients [20]. No specific information was provided in three studies [31–33] (Table 1). Three studies were funded by university or government agencies [18, 28, 30], whilst the other eight studies had no sponsorship [17, 19–21, 29, 31–33]. The main characteristics and results of included studies were summarized in Tables 2 and 3.

**Table 1:** Definition of bronchiolitis used in the studies identified.

Study	Definition	Wheezing Type
Bansal A, et al. (2010) [20]	Presence of tachypnea and either chest indrawing (any subcostal recession) or one of the following danger signs: cyanosis, inability to feed/drink, lethargy, and convulsions.	Combination of wheezing and non-wheezing patients
Deng XM, et al. (2008) [33]	Diagnosed according to the diagnostic standards stated in “Zhu Fu-tang Practical Pediatrics 7th edition” and “Pediatrics of Traditional Chinese Medicine” and patients generally presenting with fever, respiratory distress, and throat inflammation.	No information
Feng X, et al. (2006) [32]	Clinically diagnosed with symptoms of cough, episodic panting and chest oppression, emphysema and in some cases rales, wheeze, and low fever.	No information
Gupta P. (2013) [19]	Acute onset of rapid breathing with wheezing and/or crackles in a young infant with a prodromal upper respiratory catarrh.	First wheeze
Heydarian F, et al. (2011) [18]	Diagnosed clinically and radiologically as first episode of wheezing.	First wheeze
Kose M, et al. (2014) [21]	A history of preceding viral upper respiratory infection followed by wheezing and crackles on auscultation, first wheezing episode, and a clinical severity score (CSS) of 4–8 on admission. Viral respiratory infection was diagnosed on clinical grounds. The CSS was defined based on four parameters including respiratory rate, degree of wheezing, degree of accessory muscle use, and general condition.	First wheeze
Modaresi MR, et al. (2015) [28]	Acute onset of respiratory distress, positive wheezing in physical examination, a chest radiograph compatible with bronchiolitis, and respiratory distress assessment instrument (RDAI) score of at least 5.	First wheeze
Naz F, et al. (2014) [17]	A prodromal history consistent with upper respiratory tract infection followed by wheezing and/or crackles on auscultation, and a clinical severity score >4 on presentation.	First wheeze
Saad K, et al. (2015) [29]	Diagnosed by two pediatricians and defined as an acute onset lower respiratory tract symptoms for <2 weeks, with evidence of a viral infection (rhinorrhea, coryza, cough or fever), abnormal auscultatory findings (wheeze and/or crackles), and increased respiratory effort (tachypnea and intercostal retractions).	First wheeze
Shang X, et al. (2015) [30]	Clinically diagnosed with a first episode of wheezing.	First wheeze
Wang WS, et al. (1997) [31]	Clinically diagnosed by physician in the hospital and with respiratory distress.	No information

**Table 2:** Characteristics and summary of results of herbal medicine studies.

Study	Design	Participants		Interventions		Treatment duration (days)	Primary and secondary outcomes	Findings	Adverse events
		Sample size (Experimental/ Control)	Age range (months)	Experimental group	Control group				
Wang WS, et al., 1997, China [31]	Cohort study	43/23	1–24	Intravenous *Shuang Huang Lian* 60 mg/kg daily and dexamethasone	Intravenous ribavirin 10–15 mg/kg daily and dexamethasone	2	Duration of hospitalization, cure rate, and time to resolution of fever, dyspnea, and pulmonary signs	Patients treated with *Shuang Huang Lian* had shorter length of hospital stay than ribavirin group (8.92 vs. 10.70 days, MD: -1.78, 95% CI: -2.72 to -0.84; p = 0.0002). 86% (37/43) patients in the *Shuang Huang Lian* group were cured compared with 61% (14/23) in the control (RR: 1.41, 95% CI: 1.00 to 2.00; p = 0.05). Mean time to resolution of fever (19.33 vs. 28.62 hours, MD: -9.29, 95% CI: -12.81 to -5.77; p<0.00001), dyspnea (4.22 vs. 5.64 days, MD: -1.42, 95% CI: -1.93 to -0.91; p<0.00001), and pulmonary signs of bronchiolitis (6.54 vs. 8.42 days, MD: -1.88, 95% CI: -2.60 to -1.16; p<0.00001) were shorter in *Shuang Huang Lian* group.	Not reported
Feng X, et al., 2006, China [32]	Cohort study	45/30	2–24	Modified *Jie Jing Ding Chuan Zhi Xiao Tang* oral liquid 3 times daily	*Xiao Er Ke Chuan Ling* granules dissolved in boiled water 3 times daily	7	Cure rate	67% (30/45) patients in the experimental group were cured compared to 13% (4/30) in the control (RR: 5.00, 95% CI: 1.96 to 12.74; p = 0.0007).	Not reported
Deng XM, et al., 2008, China [33]	Cohort study	50/41	1–24	Aerosol inhalation of *Xiao Er Zhi Chuan Tang*, 20 ml to be inhaled via a nebulizer over 20–30 minutes twice daily in combination with conventional medicines such as third generation cephalosporins, aminophylline, and oxygen	Conventional medicines, including third generation cephalosporins, aminophylline, oxygen, digitalis, or frusemide	7	Cure rate, oxygen saturation after treatment, and time to resolution of cough, fever, dyspnea, chest wall retraction, rales, and wheezing	92% (46/50) patients in the experimental group were cured as compared with 73% (30/41) in the control (RR: 1.26, 95% CI: 1.03 to 1.54; p = 0.03). Time to resolution of cough (4.5 vs. 6.8 days, MD: -2.30, 95% CI: -3.02 to -1.58; p<0.00001), fever (3.5 vs. 5.1 days, MD: -1.60, 95% CI: -2.14 to -1.06; p<0.00001), dyspnea (2.3 vs. 4.8 days, MD: -2.50, 95% CI: -3.02 to -1.98; p<0.00001), chest wall retraction (1.2 vs. 2.8 days, MD: -1.60, 95% CI: -1.95 to -1.25; p<0.00001), rales (4.7 vs. 6.7 days, MD: -2.00, 95% CI: -2.66 to -1.34; p<0.00001), and wheezing (3.2 vs. 5.1 days, MD: -1.90, 95% CI: -2.60 to -1.20; p<0.00001) was shorter in experimental group. A significantly higher oxygen saturation was detected in the experimental group than control at 24 (93.8 vs. 90.2%, MD: 3.60, 95% CI: 2.75 to 4.45; p<0.00001), 48 (94.3 vs. 92.1%, MD: 2.20, 95% CI: 1.36 to 3.04; p<0.00001), 72 (95.7 vs. 94.1%, MD: 1.60, 95% CI: 0.96 to 2.24; p<0.00001), and 96-hour (96.4 vs. 95.0%, MD: 1.40, 95% CI: 0.76 to 2.04; p<0.0001) after treatment initiation.	Not reported
Shang X, et al., 2015, China [30]	RCT	67/66	3–24	*Laggera pterodonta* mixture (3 ml for patients younger than 12 months and 5 ml for patients aged 1–2 years, given 3 times daily	Placebo	5	Proportion of children fulfilling the discharge criteria, clinical severity scores, respiratory rate, oxygen saturation, wheezing, and fever	Higher proportion of patients in the *Laggera pterodonta* group were eligible for discharge at 96-hour (65/67 vs. 50/66, RR: 1.28, 95% CI: 1.11 to 1.48; p = 0.0007) and 120-hour (66/67 vs. 59/66, RR: 1.10, 95% CI: 1.01 to 1.20; p = 0.03) after treatment. The *Laggera pterodonta* group was associated with significantly lower clinical severity scores, respiratory rate, wheezing, and heart rate, as well as higher oxygen saturation levels. The proportion of children with fever was not significantly different between the two groups. Effect estimates were not computed for the secondary outcomes in the study due to the lack of data.	Vomiting (experimental, n = 0; control, n = 1) Diarrhea (experimental, n = 8; control, n = 11)

Deng XM, et al. (2008) [33]: Cure rate was defined as proportion of patients whose breathing recovered to normal condition, rales in lungs disappeared, X-ray examination showing disappearance or minimal inflammatory shadows, no cough and oxygen saturations returning to normal.

Feng X, et al. (2006) [32]: Cure rate was defined as proportion of patient who recovered to normal condition after a course of treatment.

Wang WS, et al. (1997) [31]: Cure rate was defined as proportion of patients who recovered to normal body temperature, looked alert, ability to feed, no cough, no other pulmonary signs and symptoms of bronchiolitis, after a course of treatment.

**Table 3:** Characteristics and summary of results of supplement studies.

Study	Design	Participants		Interventions		Treatment duration (days)	Primary and secondary outcomes	Findings	Adverse events
		Sample size (Experimental/ Control)	Age range (months)	Experimental group	Control group				
Saad K, et al., 2015, Egypt [29]	RCT	44/45	3–23	Vitamin D_3_ drops 100 IU/kg/day in combination with standard treatments (intravenous fluids, oxygen, antipyretic, salbutamol, and epinephrine)	Placebo and standard treatments (intravenous fluids, oxygen, salbutamol, and epinephrine)	7	Duration of hospitalization, time to resolution of bronchiolitis symptoms, duration of intravenous fluid therapy, and duration of oxygen therapy	Vitamin D group had a significantly shorter duration of hospitalization (139 vs. 198 hours, MD: -59.00, 95% CI: -63.66 to -54.34; p<0.00001), mean time for resolution of bronchiolitis (96 vs. 145 hours, MD: -49.00, 95% CI: -53.25 to -44.75; p<0.00001), and time for improvement of oral feeding (20 vs. 36 hours, MD: -16.00, 95% CI: -17.47 to -14.53; p<0.00001). There were no differences in time to resolution of tachypnea (70 vs. 72 hours, MD: -2.00, 95% CI: -4.71 to 0.71; p = 0.15), chest retractions (69 vs. 70 hours, MD: -1.00, 95% CI: -3.91 to 1.91; p = 0.50), duration of intravenous therapy (26 vs. 28 hours, MD: -2.00, 95% CI: -5.08 to 1.08; p = 0.20), and duration of oxygen supplementation (51 vs. 52 hours, MD: -1.00, 95% CI: -2.96 to 0.96; p = 0.32).	Diarrhea (experimental, n = 2; control, n = 2)
Naz F, et al., 2014, Pakistan [17]	RCT	50/50	2–24	Nebulized 20 mg N- acetylcysteine in 3 ml of 0.9% saline 3 times daily	Nebulized 2.5 mg salbutamol in 3 ml of 0.9% saline 3 times daily	5	Duration of hospitalization and clinical severity score	Duration of hospitalization was not significantly different between groups (4.36 vs. 4.98 days, MD: -0.62, 95% CI: -1.48 to 0.24; p = 0.16). Patients receiving N- acetylcysteine showed better improvement in clinical severity score from day 1 to day 5 of admission than those receiving salbutamol (-4.50 vs. -2.78, MD: -1.72, 95% CI: -1.87 to -1.57; p<0.0001).	None
Bansal A, et al., 2010, India [20]	RCT	11/13	2–24	Oral zinc 20 mg/day once daily	Placebo	5	Time to resolution of bronchiolitis	There was no significant difference in median time to resolution of symptoms between the two groups (24 vs. 48 hours; p = 0.58).	Not reported
Heydarian F, et al., 2011, Iran [18]	RCT	25/25	2–23	Oral zinc sulfate 1% at rate of 2 mg/kg for infants under 1 year old and 20 mg of elemental zinc for patients older than 1 year old	Placebo	3	Resolution of clinical symptoms at 24, 48, and 72 hours after treatment, and proportion of patients who showed complete remission of tachypnea, cyanosis, wheezing, intercostal, and subcostal retractions after treatment	No significant difference between the two groups in symptoms resolution. At 24, 48, and 72 hours post-admission, the proportion of patients with rhinorrhea, fever, tachypnea, dyspnea, nasal flaring, subcostal retraction, intercostal retraction, cyanosis, or wheezing was similar between groups. At 24 hours after treatment, more patients (12/25) in placebo group showed complete recovery of bronchiolitis symptoms than patients (5/25) treated with zinc (RR: 1.54, 95% CI: 1.01 to 2.35; p = 0.05).	Not reported
Gupta P., 2013, India [19]	RCT	26/74	3–24	Oral zinc 10 mg/day (infants less than 6 months) and 20 mg/day (6–24 months) once daily, in combination with standard treatments (intravenous fluids, oxygen, antipyretic, salbutamol, and epinephrine)	Placebo in combination with standard treatments (intravenous fluids, oxygen, antipyretic, salbutamol, and epinephrine)	7	Duration of hospitalization, time to resolution of severe bronchiolitis or individual signs/symptoms, intravenous therapy, and oxygenation	No significant difference in median duration of hospitalization (80, 95% CI: 64–96 vs. 96, 95% CI: 88–104 hours; p = 0.96), time to resolution of severe bronchiolitis (46, 95% CI: 33–59 vs. 40, 95% CI: 36–44 hours; p = 0.37), resolution of lower chest indrawing (46, 95% CI: 33–59 vs. 40, 95% CI: 35–45 hours; p = 0.35), fast breathing (64, 95% CI: 44–84 vs. 72, 95% CI: 62–82 hours; p = 0.90), inability to feed (16, 95% CI: 11–21 vs. 8, 95% CI: 8–8 hours; p = 0.19), duration of intravenous therapy (32, 95% CI: 28–36 vs. 32, 95% CI: 27–37 hours; p = 0.51), and duration of oxygen therapy (16, 95% CI: 10–22 vs. 16, 95% CI: 13–19 hours; p = 0.79).	Vomiting (experimental, n = 0; control, n = 1) Diarrhea (experimental, n = 0; control, n = 1)
Kose M, et al., 2014, Turkey [21]	RCT	19 (E1)/19 (E2)/18	1–24	E1: Inhaled magnesium sulfate 150 mg, diluted to 4 ml with 0.9% saline solution (2 doses) E2: Inhaled salbutamol 0.15 mg/kg and magnesium sulfate 150 mg, diluted to 4 ml with 0.9% saline solution (2 doses)	Inhaled salbutamol 0.15 mg/kg, diluted to 4 ml with 0.9% saline solution (2 doses)	1	Duration of hospitalization, clinical severity scores, and heart rate	No significant difference was observed in the mean duration of hospitalization between the groups (24 (E1) vs. 20 (E2) vs. 24 hours; p>0.05). Mean clinical severity scores was higher in magnesium sulfate group compared to salbutamol/magnesium sulfate group at 4 hours post-treatment (4.7 vs. 3.4, MD: 1.30, 95% CI: 0.66 to 1.94; p<0.0001) or compared to salbutamol group (4.7 vs. 4.0, MD: 0.70, 95% CI: 0.12 to 1.28; p = 0.02). There was no significant difference in heart rate at 4 hours post-treatment (138.2 (E1) vs. 138.4 (E2) vs. 149.4 bpm; p>0.05).	None
Modaresi MR, et al., 2015, Iran [28]	RCT	60/60	1–12	Nebulized magnesium sulfate 40 mg/kg and epinephrine 0.1 ml/kg mixed with normal saline (3 doses)	Nebulized epinephrine 0.1 ml/kg mixed with normal saline (3 doses)	1	Duration of hospitalization, clinical severity scores, duration of oxygen use, and use of respiratory support	No significant differences were detected in duration of hospitalization (84.3 vs. 84.7 hours, MD: -0.40, 95% CI: -3.94 to 3.14; p = 0.82), duration of oxygen use (10.4 vs. 11.1 hours, MD: -0.70, 95% CI: -1.70 to 0.30; p = 0.17), and use of respiratory support (2/60 vs. 3/60, RR: 0.67, 95% CI: 0.12 to 3.85; p = 0.65) between the two groups. Improvement in clinical severity scores was significantly better in patients treated with nebulized magnesium sulfate than the control group in second (-8.6 vs. -7.3, MD: -1.30, 95% CI: -1.47 to -1.13; p<0.00001) and third day (-10.3 vs. -9.3, MD: -1.00, 95% CI: -1.15 to -0.85; p<0.00001).	Not reported

### Interventions

Four herbal preparations [30–33] and four supplements [17–21, 28, 29] were examined. They included Chinese herbal medicine (3 herbal mixtures [31–33] and 1 herbal monopreparation [30]), vitamin D [29], N-acetylcysteine [17], zinc [18–20], and magnesium [21, 28]. Five studies compared placebo to treatment [18–20, 29, 30], whilst another six studies had active comparators such as ribavirin [31], salbutamol [17, 21], epinephrine [28], conventional care (cephalosporins, aminophylline, and oxygen) [33], or Chinese herbal medicine *Xiao Er Ke Chuan Ling* [32]. The most commonly reported outcomes were duration of hospital stay (n = 6 studies) [17, 19, 21, 28, 29, 31], time to symptom resolution (n = 6 studies) [18–20, 29, 31, 33], cure rate (n = 3 studies) [31–33], and clinical severity scores during hospitalization (n = 4 studies) [17, 21, 28, 30].

### Study quality assessment

#### Randomized controlled trials

**Fig 2:**
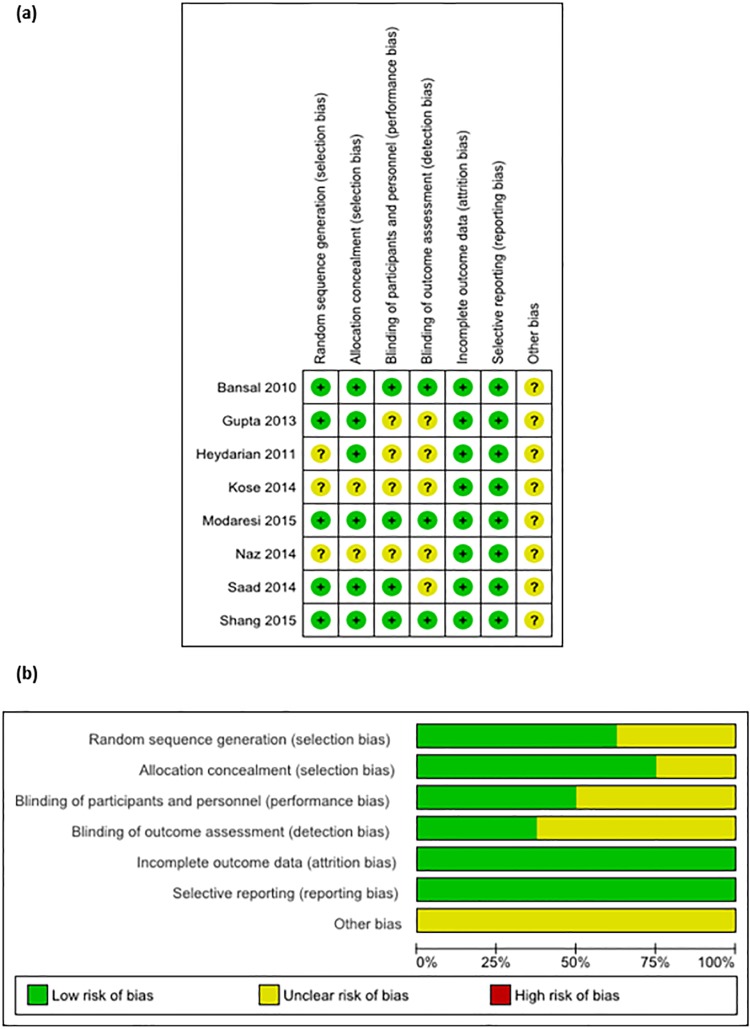
Assessment of risk of bias according to a recommended tool for randomized controlled trials by the Cochrane Handbook for Systematic Reviews of Interventions. (a) Risk of bias summary showing review authors’ judgments about each risk of bias domain for 8 randomized controlled trials. (b) Risk of bias graph showing each risk of bias domain presented as percentages across the studies.

The methodological quality of the 8 randomized controlled trials (RCTs) was generally moderate. Three trials had all domains judged as low risk of bias [20, 28, 30]. Most trials had an appropriate method of randomization as well as allocation concealment. Seven studies mentioned double blinding, of which only three described details of blinding of patients, personnel, and outcome assessors [20, 28, 30]. All studies were judged to have sufficient reporting of outcomes and have low risk of bias of selective outcome reporting. However, all studies had an unclear risk of other source of bias (Fig 2).

#### Cohort studies

The observational study by Feng and colleagues lacked methodological details for quality assessment [32]. The other two studies generally had a good quality of cohort selection and comparability, but, independent blind outcome assessment and adequacy of follow up were not described [31, 33].

### Effects of complementary and alternative medicine in bronchiolitis

#### Chinese herbal medicine

Four different preparations of Chinese herbal medicine were examined. The herbal composition and medicinal properties of the respective Chinese herbal medicine are described in S1 Table. The mechanism of action for each herbal medicine is presented in S2 Table.

#### Shuang Huang Lian

In the cohort study by Wang et al. (n = 66), the authors examined the use of *Shuang Huang Lian* injection and found a significant decrease in the length of hospital stay (MD: -1.78 days, 95% CI: -2.72 to -0.84; p<0.01) and duration of pulmonary signs of bronchiolitis (MD: -1.88 days, 95% CI: -2.60 to -1.16; p<0.01) compared with patients receiving ribavirin [31]. The study also examined other outcomes as shown in Table 2.

#### Laggera pterodonta

The randomized trial examining *Laggera pterodonta* did not assess for duration of hospitalization. The study (n = 133) showed that 97% of patients were eligible for discharge on the third day of admission compared to 76% in the placebo group (RR: 1.28, 95% CI: 1.11 to 1.48; p<0.01). The study also showed a significantly lower clinical severity score throughout hospital stay among the patients receiving *Laggera pterodonta* (effect estimates were not calculated due to the lack of data in the original study) [30]. The study also examined other outcomes as shown in Table 2.

#### *Ephedra*-containing herbal decoction

The following two studies did not evaluate the outcome of hospital length of stay.

In the first cohort study by Feng et al. (n = 75), the authors found 54% higher cure rate in patients administered the oral liquid *Jie Jing Ding Chuan Zhi Xiao Tang* formulation (RR: 5.00, 95% CI: 1.96 to 12.74; p<0.01) [32].

The other cohort study (n = 91) examined the effects of inhaled *Xiao Er Zhi Chuan Tang* as an adjuvant to usual care. The herbal formulation was found to shorten the duration of respiratory symptoms such as cough (MD: -2.30 days; 95% CI: -3.02 to -1.58; p<0.01), fever (MD: -1.60 days; 95% CI: -2.14 to -1.06; p<0.01), dyspnea (MD: -2.50 days, 95% CI: -3.02 to -1.98; p<0.01), chest wall retraction (MD: -1.60 days, 95% CI: -1.95 to -1.25; p<0.01), rales (MD: -2.00 days, 95% CI: -2.66 to -1.34; p<0.01), and wheezing (MD: -1.90 days, 95% CI: -2.60 to -1.20; p<0.01) and result in 20% higher cure rate (RR: 1.26, 95% CI: 1.03 to 1.54; p = 0.03) compared to conventional treatment [33]. The study also examined other outcomes as shown in Table 2.

#### Supplements

The mechanism of action for each supplement is shown in S2 Table.

#### Vitamin D

One randomized trial (n = 89) examined the effects of vitamin D supplementation in bronchiolitis. The authors found vitamin D was more effective than placebo in reducing hospital length of stay (MD: -59.00 hours, 95% CI: -63.66 to -54.34; p<0.01), duration of bronchiolitis symptoms (MD: -49.00 hours, 95% CI: -53.25 to -44.75; p<0.01), and duration of feeding problem (MD: -16.00 hours, 95% CI: -17.47 to -14.53; p<0.01) [29]. The study also examined other outcomes as shown in Table 3.

#### N-acetylcysteine

In the randomized trial (n = 100) by Naz and colleagues, N-acetylcysteine showed no significant benefit in length of hospital stay (MD: -0.62 days, 95% CI: -1.48 to 0.24; p = 0.16) compared to salbutamol. Patients receiving N- acetylcysteine showed better improvement in clinical severity score than those receiving salbutamol (MD: -1.72, 95% CI: -1.87 to -1.57; p<0.0001) [17].

#### Zinc

In a randomized trial (n = 100) conducted by Gupta and collaborators, oral zinc was reported to have no beneficial effect in the outcome of length of hospital stay [19]. Together with two other trials, zinc showed no benefit in managing clinical symptoms of bronchiolitis (Table 3) [18–20]. The latter two studies did not assess for the outcome of length of hospital stay.

#### Magnesium

Two studies evaluated magnesium, either alone or as an adjuvant therapy for bronchiolitis in infants. The study by Modaresi and co-workers (n = 120) found no significant difference in duration of hospitalization (MD: -0.40, 95% CI: -3.94 to 3.14; p = 0.82) between magnesium-treated patients and conventional treatment group. However, magnesium treatment was associated with significantly better improvement in clinical severity scores compared to epinephrine (Table 3) [28].

In a three-arm randomized trial by Kose et al. (n = 56), no significant difference was observed in the duration of hospitalization between the groups (20 (magnesium/salbutamol) vs. 24 (magnesium) vs. 24 hours (salbutamol); p>0.05). Inhalation of magnesium and salbutamol combination resulted in lower clinical severity scores compared to those treated with salbutamol (MD: -0.60, 95% CI: -1.18 to -0.02; p = 0.04) or magnesium alone (MD: -1.30, 95% CI: -1.94 to -0.66; p<0.01) [21].

#### Adverse events

Five of the total eleven studies provided information on adverse events (AE) [17, 19, 21, 29, 30]. Kose et al. reported no AE such as hypotension, arrhythmias, and loss of deep tendon reflexes in patients treated with inhaled magnesium sulfate monotherapy, salbutamol monotherapy, and salbutamol/magnesium sulfate combination therapy [21]. Likewise, Naz et al. noted that patients treated with inhaled N-acetylcysteine experienced no side effects, including stomatitis, rhinorrhea, nausea, and gastrointestinal disturbances [17]. Two studies examining oral *Laggera pterodonta* mixture [30] and oral zinc suspension [19] reported higher incidence of vomiting and diarrhea in the placebo group. The other one study reported no difference in the incidence of diarrhea between infants receiving oral vitamin D drops and placebo [29].

## Discussion

To our best knowledge, this is the first systematic review that examined the effectiveness and safety of complementary and alternative medicine (CAM) for the treatment of bronchiolitis. Our review found that two studies reported significant benefit associated with the use of CAM in the primary outcome of length of hospital stay. The first study (n = 66) was a cohort study conducted by Wang et al. and reported that Chinese herbal medicine (*Shuang Huang Lian*) reduced duration of hospitalization by 1.8 days compared to ribavirin [31]. The second study (n = 89) was a randomized controlled trial undertaken by Saad et al. and demonstrated that vitamin D decreased length of stay by 2.5 days compared to placebo [29]. On the contrary, N-acetylcysteine, zinc, and magnesium had no benefit on the length of hospital stay [17, 19, 21, 28]. Studies of Chinese herbal medicine, vitamin D, N-acetylcysteine, and magnesium showed some benefits with respect to clinical severity scores [17, 21, 28] [30], oxygen saturation [30, 33], and symptom resolution [29–31, 33]. Nonetheless, findings from the primary studies should be interpreted cautiously, given the heterogeneity across trials in the CAM treatment studied, outcome reporting, and other differences in trial design and conduct, as well as insufficient, and in some cases lack of rigorous, evidence for the different interventions (e.g., most of the studies of herbal medicine were cohort studies and there was only single study for many of the CAM interventions, of which most had a small sample size).

The current American Academy of Pediatrics (AAP) guidelines recommend that apart from length of hospital stay, parent satisfaction and patient-centered outcomes should be integrated in evaluating the effectiveness of pharmacotherapy for bronchiolitis [6]. No study identified in this review reported a patient-centered outcome. Instead, a number of studies, especially those which examined herbal decoctions used “cure rate” or a global assessment as an outcome. It was uncertain how these criteria were established, but the definitions were not uniform across studies. Furthermore, there is currently no universally validated bronchiolitis scoring system in clinical practice. In our review, Kose et al. [21] used the clinical severity score (CSS) tool developed by Wang et al. [34], whereas Modaresi et al. [28] used respiratory distress assessment instrument (RDAI) as a measure [35]. Albeit these two studies analogously examined magnesium, the use of different clinical severity scoring tools for predicting the course of illness may affect the treatment outcomes due to the dissimilar parameters being assessed.

Variability in measuring and reporting outcomes across studies of bronchiolitis precludes a formal meta-analysis. Our findings provide further evidence of inconsistency and problems with outcome reporting that can be a source of bias in this literature. There is a need to assess outcomes across the CAM studies. However, systematic assessment of treatment efficacy is often complicated by the wide range of outcome measures used by investigators. Therefore, it is necessary to agree on a core outcome set to be measured and reported in future studies assessing the impact of CAM on bronchiolitis. The Agency for Healthcare Research and Quality (AHRQ) recommends future studies to ascertain clinically relevant outcomes, particularly rates of hospitalization, need for more intensive services in the hospital, costs of care, parental satisfaction with treatment, and development of longer-term respiratory problems to facilitate clarity in communication about outcomes and increase the value of existing and future data [36]. Also of potential concern, the findings in this review may be subject to publication bias which is often difficult to detect in systematic reviews with few studies. Due to the heterogeneity in the outcomes assessed, the method of reporting, and the various types of CAM used, assessment of publication bias was not performed. Hence, we were unable to rule out the presence of publication bias in this body of literature.

There was a scarcity of information concerning the quality as well as the complete constituents of the herbal preparations used. A key reason could be the difficulty in standardization of herbal preparations. The manifold constituent nature, age of plants, harvest season, source location, and technique of crude preparation may affect the chemical composition and potency of herb derivatives [37]. Although some manufacturers have commenced to standardize herbal products, there is still a general lack of consistency in the market. On the other hand, research on supplements has been encumbered by the absence of reliable assays to detect micronutrient deficiencies. For instance, the emergence of vitamin D assays from the preliminary competitive protein binding assays through to immunoassay and liquid chromatography-tandem mass spectrometry assay have shown numerous analytical challenges. Immunoassay is the most popular method in consideration of ease of use, cost, and rapidity, however, the accuracy and specificity remain an issue of discussion [38]. The non-existence of reliable assays added challenges in elucidating nutrient-related conditions, theories, pharmacodynamics, and pharmacokinetics of mineral supplements in bronchiolitis.

Another issue was the lack of adverse events reporting. Less than half of the included studies reported adverse events, of which, two reported no adverse events [17, 21]. The adverse events documented were gastrointestinal problems associated with the use of *Laggera pterodonta* mixture, vitamin D, and zinc. This scarcity of reporting can be considered as reporting bias and as such could have underestimated the magnitude of a problem. There is empirical evidence indicating that adverse events are inadequately reported in CAM trials [39]. It is necessary to improve such reporting of harms to enable accurate, objective, and comprehensive safety data to be given to users even when there have been no observed adverse events.

Potential and theoretical adverse effects may stem from biologically active constituents from herbs, side effects of contaminants, cross-allergenicity, and CAM-drug interactions. For example, three herbal preparations [32, 33] located in this review contained *Ephedra herba*, which had the active ingredient ephedrine. Some of the common side effects of ephedrine included nausea, vomiting, psychiatric or autonomic symptoms, and palpitations [40], but none of these was reported in the included studies. Therefore, further studies on safety of these preparations are warranted, especially pertaining to CAM-drug interactions as well as risk-benefit assessments.

Thus far, we did not find other evidence from systematic reviews or meta-analyses of CAM for bronchiolitis. A Cochrane review has examined the therapeutic effect of traditional Chinese herbal medicines commonly used in China for acute bronchitis [41]. Nevertheless, our findings are consistent with the Cochrane review in the aspects of sparsity of available evidence and the need for future well-designed RCTs with adequate power in order to formulate a clinical conclusion on the use of any CAM.

### Strengths and limitations

The strengths of this review include literature search using five relevant electronic databases without language restriction. There are several limitations that must be addressed. Most of the studies had shortcomings in their study design and outcome reporting. Moreover, the presence of reporting bias may be a cause for concern as all of the current studies are published in Asia, which has previously been shown to produce a large proportion of studies with positive outcomes [42]. Outcomes reported in the primary studies were not ascertained according to the disease severity, which may have impacted the effectiveness outcomes. The study by Deng et al. also compared Chinese herbal medicine with third generation cephalosporins, aminophylline, oxygen, digitalis, or frusemide [33], suggesting the presence of secondary bacterial infection. However, we did not find any evidence of signs and diagnosis of bacterial infection in the article. Furthermore, a large proportion of the included studies (n = 5, 45%) did not report for the primary outcome of interest in this systematic review, albeit they are all inpatient studies. This may be an indication of selective outcome reporting with potential risk of bias.

In addition, publication bias has been recognized as a common phenomenon in clinical literature, in which trials with positive findings have a better chance of being published [43]. As such, any conclusions made exclusively based on published studies can be misleading. The inclusion of only published articles in this review may overestimate the potential benefit of CAM interventions, and thus we urge caution in its interpretation. Another limitation was that the review did not search for grey literature, including conference abstracts, contact with experts and trials registries. As such, we may have missed some studies. In relation to this limitation, we recommend a judicious interpretation of the evidence synthesized in this review as there may be unpublished studies with negative results that we did not include, and thus overestimating the effectiveness of CAM interventions based on the studies that were identified and included. In view of the sparsity of evidence in this area, we recommend that negative and inconclusive as well as positive results should be published or otherwise made publicly available so that the literature can provide evidence base for clinical decision-making. This will facilitate clinicians and patients to understand treatments which are effective, ineffective, safe or even harmful.

### Implications

It is very likely that more parents will use CAM for their children and this paradigm will continue to rise, particularly in diseases with few therapeutic options available [13, 44]. Current evidence from clinical studies is scarce and often not methodologically robust. Hence, future studies should direct the attention to issues identified in this review, including randomization, blinding, sample size, and complete reporting of safety profiles, particularly, the adverse effects on body systems. Randomized controlled trials would entail closer observation of patients, which would usually translate into better quality of care than would normally occur in the general population. Well-designed randomized controlled trials are considered as the gold standards for judging the benefits of an intervention and would provide the highest level of evidence.

As interest in the potential benefit of CAM grows, it is becoming imperative for healthcare providers to monitor the progress of the clinical literature and to communicate these findings to patients. Until more compelling evidence is available, physicians should remain judicious regarding the use of CAM in bronchiolitis.

## Conclusions

This review identified 11 studies evaluating the effectiveness and safety of a variety of CAM interventions among inpatients with bronchiolitis. Five studies did not examine our primary outcome of hospital length of stay. Four of the remaining six studies did not find a significant benefit associated with the primary outcome. Preliminary evidence indicated that Chinese herbal medicine mixtures, vitamin D, N-acetylcysteine, and magnesium might be potentially useful in managing the symptoms of bronchiolitis. However, the evidence was not sufficient or rigorous enough to formulate recommendations for the use of any CAM. Among studies that reported adverse events, no serious harms were noted. There is a need to conduct more high quality studies to better understand the effectiveness as well as safety of CAM for bronchiolitis in infants.

## Supporting information

S1 Table Overview ingredients of Chinese herbal medicine. (DOCX)

S2 Table Mechanism of action of herbal medicine and supplements in bronchiolitis. (DOCX)

S3 Table List of full-text articles excluded for evidence synthesis. (DOCX)

S4 Table PRISMA checklist—Preferred Reporting Items for Systematic Reviews and Meta-Analyses checklist. (DOCX)

S1 Text Database search strategy. (DOCX)
