# Supplementary material for: Complementary and alternative medicine for the treatment of bronchiolitis in infants: A systematic review
Source: PLoS One. 2017 Feb 17;12(2):e0172289. doi: 10.1371/journal.pone.0172289 (PMC5315308; doi:10.1371/journal.pone.0172289)
Supplement: S1 Table — (DOCX) [file pone.0172289.s001.docx]

**S1 Table. Overview ingredients of Chinese herbal medicine.**

| **Chinese herbal medicine** | **Main ingredients** | **Medicinal functions** |
| --- | --- | --- |
| *Jie Jing Ding Chuan Zhi Xiao Tang* [32] | *Fructus perillae frustescentis* | Anti-inflammatory and immune-regulatory effects |
|  | *Pheretima* | Clears heat and calms wheezing |
|  | *Peucedani radix* | Expels phlegm |
|  | *Chelidonii herba* | Relieves cough |
|  | *Ephedra herba* | Calms wheezing and relieves cough |
|  | *Armenicae amarum semen* | Clears lung-heat and controls cough |
|  | *Gingko semen* | Stops phlegmatic dyspnea and cough |
|  | *Scorpio* | Stops tremors and convulsions |
|  | *Dictamni cortex* | Clears heat |
|  | *Batryticatus bombyx* | Clears phlegm |
|  | *Belamcanda chinensis* | Alleviates sore throat, relieves swelling, clears lung heat, and dispels phlegm |
|  | *Farferae flos* | Resolves phlegm and cough |
| *Laggera pterodonta* mixture [30] | *Asteraceae* | Anti-inflammatory, antibacterial, and antiviral effects |
| *Shuang Huang Lian* (intravenous preparation) [31] | *Lonicerae flos* | Antipyretic, antiviral, antimicrobial against bacteria and fungi, anti-inflammation, anti-nociceptive, anti-diabetic, anti-tumor, antioxidant, anti-angiogenic, and hepatoprotective |
|  | *Scutellaria baicalensis* | Antipyretic, anti-inflammation, antibacterial, hepatoprotective, antihypertensive, and diuretic |
|  | *Forsythia suspensa* | Antiviral, antifungal, antibacterial, anti-inflammation, choleretic, antipyretic, hepatoprotective, antiemetic, and diuretic |
| *Xiao Er Ke Chuan Ling* [32] | *Ephedra herba* | Calms wheezing and relieves cough |
|  | *Gypsum fibrosum* | Relieves dyspnea and cough due to lung-heat |
|  | *Armenicae amarum semen* | Clears lung-heat and controls cough |
|  | *Trichosanthis fructus* | Relieves cough and dyspnea due to heat-phlegm |
|  | *Isatidix radix* | Clears heat from lungs and treats swollen throat |
|  | *Lonicerae flos* | Antipyretic, antiviral, antimicrobial against bacteria and fungi, anti-inflammation, anti-nociceptive, anti-diabetic, anti-tumor, antioxidant, anti-angiogenic, and hepatoprotective |
|  | *Glycyrrhizae radix* | Expels phlegm and controls cough |
| *Xiao Er Zhi Chuan Tang* [33] | *Ephedra herba* | Calms wheezing and relieves cough |
|  | *Asarum sieboldii* | Disperses wind-cold exopathogens and relieves colds and stuffy nose |
|  | *Pinellia ternata* | Relieves cough and dyspnea due to excessive phlegm |
|  | *Armenicae amarum semen* | Clears lung-heat and controls cough |
|  | *Houttuyniae herba* | Clears heat, relieves toxicity, reduces swellings, and expels phlegm |
|  | *Schisandra chinensis* | Stops cough, asthma, and pulmonary inflammation |
|  | *Sinapsis alba* | Treats cough and dyspnea due to cold-phlegm |
|  | *Lepidium apetalum* | Relieves cough, asthma, and excessive phlegm |
|  | *Belamcanda chinensis* | Alleviates sore throat, relieves swelling, clears lung heat, and dispels phlegm |
|  | *Cryptotympana pustulata fabricius* | Treats infection, sore throat, and cough |
