# Supplementary material for: Complementary and alternative medicine for the treatment of bronchiolitis in infants: A systematic review
Source: PLoS One. 2017 Feb 17;12(2):e0172289. doi: 10.1371/journal.pone.0172289 (PMC5315308; doi:10.1371/journal.pone.0172289)
Supplement: S2 Table — (DOCX) [file pone.0172289.s002.docx]

**S2 Table. Mechanism of action of herbal medicine and supplements in bronchiolitis.**

| **Complementary and alternative medicine** | **Mechanism of action** |
| --- | --- |
| *Shuang Huang Lian* | *Shuang Huang Lian* injection, a Chinese herbal intravenous preparation extracted from *Lonicerae flos* (honeysuckle), *Radix Scutellariae baicalensis*, and *Fructus Forsythiae suspensae*, is widely used to treat respiratory tract infections in China [[1](#_ENREF_1)]. The main components consist of chlorogenic acid, baicalin, and forsythia glycosides [[2](#_ENREF_2)], which have been found to have anti-inflammatory, immunomodulatory, and antiviral properties [[3-5](#_ENREF_3)]. In vitro and in vivo models have demonstrated that *Shuang Huang Lian* has an antiviral effect against respiratory syncytial virus [[6](#_ENREF_6)]. In a review by Zhang et al, the authors reported that *Shuang Huang Lian* injection could increase NK cell activity, promote production of alpha-interferon, enhance rate of lymphocyte transformation, and reduce level of CD4+ cells and ratio of CD4+/CD8+, whilst increasing CD8+ [[7](#_ENREF_7)]. |
| *Laggera pterodonta* | *Laggera pterodonta* is a common traditional herbal remedy used for over 300 years and a mixture of the aqueous extract of this medicine has been prescribed for more than 20 years in the southwest of China, particularly in Yunnan province [[8](#_ENREF_8)]. The aqueous extracts of *Laggera pterodonta* have been shown to exhibit better antiviral effects against respiratory syncytial virus compared to ribavirin in vitro [[9](#_ENREF_9)]. They also have anti-inflammatory properties in various in vivo models of both acute and chronic inflammation [[10](#_ENREF_10)]. |
| *Jie Jing Ding Chuan Zhi Xiao Tang* oral liquid (modified formula) and *Xiao Er Ke Chuan Ling* granules | Both formulations contained *Ephedra herba* which is shown to have antitussive effect against cough in animal models [[11](#_ENREF_11)]. |
| Vitamin D | The therapeutic effect of vitamin D in bronchiolitis stems from its ability to inhibit pulmonary inflammatory responses while enhancing host immune response by inducing cathelicidins, a group of antimicrobial peptides produced by neutrophils, macrophages, and epithelial cells [[12](#_ENREF_12)]. |
| N-acetylcysteine | N-acetylcysteine is a supplement that acts as a precursor to produce glutathione, a vital cellular antioxidant in the body. The anti-inflammatory, antioxidant and mucokinetic properties of N-acetylcysteine play a role in respiratory conditions [[13-15](#_ENREF_13)]. |
| Zinc | Zinc is an antioxidant and a crucial trace element in the body that modulates cellular function of immune system and anti-inflammatory response, suggesting a potential treatment for acute and chronic inflammation of respiratory tract [[16](#_ENREF_16)]. |
| Magnesium | Magnesium is the body’s second most plentiful mineral that plays a role in protein synthesis, neuromuscular conduction, and immune mechanism. In addition to bronchodilating effect, supplementation with magnesium is safe and beneficial for improving pulmonary function and severe acute asthma [[17](#_ENREF_17), [18](#_ENREF_18)]. |

**References**

1. Zhang J, Shang H, Zheng W, Hu J, Xu H, Wang H, et al. Systematic review on the compatibility of Shuanghuanglian injection combined with western medical injections. Journal of Evidence-Based Medicine. 2010;3(1):27-36. doi: 10.1111/j.1756-5391.2010.01068.x.

2. Zhou W, Di L, Bi X, Chen L, Du Q. Study on in situ intestinal absorption of active ingredients in Shuanghuanglian oral liquid in rats. China journal of Chinese Materia Medica. 2011;36(13):1733-8. Epub 2011/10/29. PubMed PMID: 22032135.

3. Wang GF, Shi LP, Ren YD, Liu QF, Liu HF, Zhang RJ, et al. Anti-hepatitis B virus activity of chlorogenic acid, quinic acid and caffeic acid in vivo and in vitro. Antiviral Res. 2009;83(2):186-90. Epub 2009/05/26. doi: 10.1016/j.antiviral.2009.05.002. PubMed PMID: 19463857.

4. Chu ZY, Chu M, Teng Y. Effect of baicalin on in vivo anti-virus. China Journal of Chinese Materia Medica. 2007;32(22):2413-5. Epub 2008/02/09. PubMed PMID: 18257272.

5. Li H, Wu J, Zhang Z, Ma Y, Liao F, Zhang Y, et al. Forsythoside a inhibits the avian infectious bronchitis virus in cell culture. Phytotherapy Research : PTR. 2011;25(3):338-42. Epub 2010/08/03. doi: 10.1002/ptr.3260. PubMed PMID: 20677175.

6. Wang YH, Xu KJ, Jiang WS. Experimental and clinical study of shuanghuanglian aerosol in treating acute respiratory tract infection. Chinese Journal of Integrated Traditional and Western Medicine. 1995;15(6):347-50. Epub 1995/06/01. PubMed PMID: 7549385.

7. Zhang H, Chen Q, Zhou W, Gao S, Lin H, Ye S, et al. Chinese Medicine Injection Shuanghuanglian for Treatment of Acute Upper Respiratory Tract Infection: A Systematic Review of Randomized Controlled Trials. Evidence-based Complementary and Alternative Medicine : eCAM. 2013;2013:987326. doi: 10.1155/2013/987326. PubMed PMID: PMC3625553.

8. Jiangsu New Medical College. A Dictionary of Traditional Chinese Drugs. 1st edition ed. Shanghai: Shanghai People’s Press; 1977.

9. Li Y, Ooi LS, Wang H, But PP, Ooi VE. Antiviral activities of medicinal herbs traditionally used in southern mainland China. Phytotherapy Research : PTR. 2004;18(9):718-22. Epub 2004/10/13. doi: 10.1002/ptr.1518. PubMed PMID: 15478204.

10. Wu Y, Zhou C, Li X, Song L, Wu X, Lin W, et al. Evaluation of antiinflammatory activity of the total flavonoids of Laggera pterodonta on acute and chronic inflammation models. Phytotherapy Research : PTR. 2006;20(7):585-90. Epub 2006/05/05. doi: 10.1002/ptr.1918. PubMed PMID: 16673449.

11. Minamizawa K, Goto H, Shimada Y, Terasawa K, Haji A. Effects of eppikahangeto, a Kampo formula, and Ephedrae herba against citric acid-induced laryngeal cough in guinea pigs. Journal of Pharmacological Sciences. 2006;101(2):118-25. doi: 10.1254/jphs.FP0060135.

12. Hughes DA, Norton R. Vitamin D and respiratory health. Clinical and Experimental Immunology. 2009;158(1):20-5. Epub 2009/09/10. doi: 10.1111/j.1365-2249.2009.04001.x. PubMed PMID: 19737226; PubMed Central PMCID: PMC2759054.

13. Mata M, Morcillo E, Gimeno C, Cortijo J. N-acetyl-L-cysteine (NAC) inhibit mucin synthesis and pro-inflammatory mediators in alveolar type II epithelial cells infected with influenza virus A and B and with respiratory syncytial virus (RSV). Biochemical Pharmacology. 2011;82(5):548-55. Epub 2011/06/04. doi: 10.1016/j.bcp.2011.05.014. PubMed PMID: 21635874.

14. Mata M, Sarrion I, Armengot M, Carda C, Martinez I, Melero JA, et al. Respiratory syncytial virus inhibits ciliagenesis in differentiated normal human bronchial epithelial cells: effectiveness of N-acetylcysteine. PLoS One. 2012;7(10):e48037. Epub 2012/11/03. doi: 10.1371/journal.pone.0048037. PubMed PMID: 23118923; PubMed Central PMCID: PMC3485262.

15. University of Maryland Medical Center. Complementary and Alternative Medicine Guide: Supplement Cysteine. Baltimore, Maryland, USA [updated June 26, 2014]. Available from: http://umm.edu/health/medical/altmed/supplement/cysteine.

16. Morgan CI, Ledford JR, Zhou P, Page K. Zinc supplementation alters airway inflammation and airway hyperresponsiveness to a common allergen. J Inflamm. 2011;8:36. Epub 2011/12/14. doi: 10.1186/1476-9255-8-36. PubMed PMID: 22151973; PubMed Central PMCID: PMC3250936.

17. Guerrera MP, Volpe SL, Mao JJ. Therapeutic uses of magnesium. American Family Physician. 2009;80(2):157-62. Epub 2009/07/23. PubMed PMID: 19621856.

18. Mathew R, Altura BM. The role of magnesium in lung diseases: asthma, allergy and pulmonary hypertension. Magnes Trace Elem. 1991;10(2-4):220-8. Epub 1991/01/01. PubMed PMID: 1844555.
