# Supplementary material for: Complementary and alternative medicine for the treatment of bronchiolitis in infants: A systematic review
Source: PLoS One. 2017 Feb 17;12(2):e0172289. doi: 10.1371/journal.pone.0172289 (PMC5315308; doi:10.1371/journal.pone.0172289)
Supplement: S3 Table — (DOCX) [file pone.0172289.s003.docx]

**Appendix 2. List of full-text articles excluded for evidence synthesis.**

| **No.** | **Study** | **Title** | **Reason for exclusion** |
| --- | --- | --- | --- |
| 1. | Hanifa, et al. (2011) | Vitamin D and acute respiratory tract infection | Review |
| 2. | Hageman, et al. (2012) | The impact of dietary long-chain polyunsaturated fatty acids on respiratory illness in infants and children | Review |
| 3. | Oliveira, et al. (2016) | Exogenous glutamine in respiratory diseases: Myth or reality? | Review |
| 4. | Roqué i Figuls, et al. (2016) | Chest physiotherapy for acute bronchiolitis in paediatric patients between 0 and 24 months old | Review |
| 5. | Enriquez, et al. (2012) | Nebulised deoxyribonuclease for viral bronchiolitis in children younger than 24 months | Review |
| 6. | Kjolhede, et al. (1995) | Clinical trial of vitamin A as adjuvant treatment for lower respiratory tract infections | Results were not stratified to bronchiolitis |
| 7. | Liu, et al. (1997) | Effects of selenium supplement on acute lower respiratory tract infection caused by respiratory syncytial virus | Results were not stratified to bronchiolitis |
| 8. | Kong, et al. (1993) | Treatment of acute bronchiolitis with Chinese herbs | Results were not stratified to age group specified in inclusion criteria |
| 9. | Atwell, et al. (2013) | Respiratory hospitalisation of infants supplemented with docosahexaenoic acid as preterm neonates | CAM as prophylaxis |
| 10. | Lapillonne, et al. (2014) | Infants fed formula with added long chain polyunsaturated fatty acids have reduced incidence of respiratory illnesses and diarrhea during the first year of life | CAM as prophylaxis |
| 11. | Pastor, et al. (2006) | Infants fed docosahexaenoic acid- and arachidonic acid-supplemented formula have decreased incidence of bronchiolitis/bronchitis the first year of life | CAM as prophylaxis |
| 12. | Pinnock, et al. (1988) | Vitamin A status of children with a history of respiratory syncytial virus infection in infancy | CAM as prophylaxis |
| 13. | Inamo, et al. (2011) | Serum vitamin D concentrations and associated severity of acute lower respiratory tract infections in Japanese hospitalized children | CAM as prophylaxis |
| 14. | Veeranki, et al. (2014) | Association of folic acid supplementation during pregnancy and infant bronchiolitis | CAM as prophylaxis |
| 15. | Leis, et al. (2012) | Vitamin D intake in young children with acute lower respiratory infection | CAM as prophylaxis |
| 16. | Gajdos, et al. (2010) | Effectiveness of chest physiotherapy in infants hospitalized with acute bronchiolitis: a multicenter, randomized, controlled trial | No CAM tested in the study |
| 17. | Livni, et al. (2010) | A randomized, double-blind study examining the comparative efficacies and safety of inhaled epinephrine and nasal decongestant in hospitalized infants with acute bronchiolitis | No CAM tested in the study |
| 18. | Flores, et al. (2016) | A randomized trial of nebulized 3% hypertonic saline with salbutamol in the treatment of acute bronchiolitis in hospitalized infants | No CAM tested in the study |
| 19. | Pinto, et al. (2016) | Duration of hospitalization in association with type of inhalation therapy used in the management of children with nonsevere, acute bronchiolitis | No CAM tested in the study |
| 20. | Tibby, et al. (2000) | Exogenous surfactant supplementation in infants with respiratory syncytial virus bronchiolitis | No CAM tested in the study |
