# Supplementary material for: Complementary and alternative medicine for the treatment of bronchiolitis in infants: A systematic review
Source: PLoS One. 2017 Feb 17;12(2):e0172289. doi: 10.1371/journal.pone.0172289 (PMC5315308; doi:10.1371/journal.pone.0172289)
Supplement: S1 Text — (DOCX) [file pone.0172289.s005.docx]

**S1 Text. Database search strategy.**

**Database: MEDLINE Search Strategy:**

1. “alternative medicine”
2. “complementary medicine”
3. “herbal medicine”
4. herb*
5. “traditional medicine”
6. “chinese medicine”
7. “chinese traditional”
8. “asian traditional”
9. “alternative therapy”
10. “complementary therapy”
11. “herbal therapy”
12. “traditional therapy”
13. supplement*
14. antioxidant
15. vitamin
16. natural
17. homeopathy
18. kinesiology
19. kinesiotherapy
20. “osteopathic medicine”
21. “osteopathic manipulation”
22. #1 or #2 or #3 or #4 or #5 or #6 or #7 or #8 or #9 or #10 or #11 or #12 or #13 or #14 or #15 or #16 or #17 or #18 or #19 or #20 or #21
23. bronchiolitis
24. #22 and #23
25. Filter: Publication date to June 30, 2016

**Database: Embase Search Strategy:**

1. alternative medicine/
2. complementary medicine.mp.
3. herbal medicine/
4. herb*.mp.
5. traditional medicine/
6. chinese medicine/
7. chinese traditional.mp.
8. asian traditional.mp.
9. alternative therapy.mp.
10. complementary therapy.mp.
11. herbal therapy.mp.
12. traditional therapy.mp.
13. supplement*.mp.
14. antioxidant/
15. vitamin/
16. natural.mp.
17. homeopathy/
18. kinesiology/
19. kinesiotherapy/
20. osteopathic medicine/
21. osteopathic manipulation.mp.
22. 1 or 2 or 3 or 4 or 5 or 6 or 7 or 8 or 9 or 10 or 11 or 12 or 13 or 14 or 15 or 16 or 17 or 18 or 19 or 20 or 21
23. bronchiolitis/
24. 22 and 23

**Database: CINAHL Search Strategy:**

1. “alternative medicine”
2. “complementary medicine”
3. “herbal medicine”
4. herb*
5. “traditional medicine”
6. “chinese medicine”
7. “chinese traditional”
8. “asian traditional”
9. “alternative therapy”
10. “complementary therapy”
11. “herbal therapy”
12. “traditional therapy”
13. supplement*
14. antioxidant
15. vitamin
16. natural
17. homeopathy
18. kinesiology
19. kinesiotherapy
20. “osteopathic medicine”
21. “osteopathic manipulation”
22. S1 or S2 or S3 or S4 or S5 or S6 or S7 or S8 or S9 or S10 or S11 or S12 or S13 or S14 or S15 or S16 or S17 or S18 or S19 or S20 or S21
23. bronchiolitis
24. S22 and S23

**Database: AMED Search Strategy:**

1. alternative medicine.mp.
2. complementary medicine.mp.
3. herbal medicine.mp.
4. herb*.mp.
5. traditional medicine.mp.
6. chinese medicine.mp.
7. chinese traditional.mp.
8. asian traditional.mp.
9. alternative therapy.mp.
10. complementary therapy.mp.
11. herbal therapy.mp.
12. traditional therapy.mp.
13. supplement*.mp.
14. antioxidant.mp.
15. vitamin.mp.
16. natural.mp.
17. homeopathy.mp.
18. kinesiology.mp.
19. kinesiotherapy.mp.
20. osteopathic medicine.mp.
21. osteopathic manipulation.mp.
22. 1 or 2 or 3 or 4 or 5 or 6 or 7 or 8 or 9 or 10 or 11 or 12 or 13 or 14 or 15 or 16 or 17 or 18 or 19 or 20 or 21
23. bronchiolitis.mp.
24. 22 and 23

**Database: CENTRAL Search Strategy:**

1. “alternative medicine”:ti,ab,kw

2. “complementary medicine”:ti,ab,kw

3. “herbal medicine”:ti,ab,kw

4. herb*:ti,ab,kw

5. “traditional medicine”:ti,ab,kw

6. “chinese medicine”:ti,ab,kw

7. “chinese traditional”:ti,ab,kw

8. “asian traditional”:ti,ab,kw

9. “alternative therapy”:ti,ab,kw

10. “complementary therapy”:ti,ab,kw

11. “herbal therapy”:ti,ab,kw

12. “traditional therapy”:ti,ab,kw

13. supplement*:ti,ab,kw

14. antioxidant:ti,ab,kw

15. vitamin:ti,ab,kw

16. natural:ti,ab,kw

17. homeopathy:ti,ab,kw

18. kinesiology:ti,ab,kw

19. kinesiotherapy:ti,ab,kw

20. “osteopathic medicine”:ti,ab,kw

21. “osteopathic manipulation”:ti,ab,kw

22. #1 or #2 or #3 or #4 or #5 or #6 or #7 or #8 or #9 or #10 or #11 or #12 or #13 or #14 or #15 or #16 or #17 or #18 or #19 or #20 or #21

23. bronchiolitis:ti,ab,kw

24. #22 and #23
